# A sociology of public responses to hospital change and closure

**DOI:** 10.1111/1467-9566.12896

**Published:** 2019-04-08

**Authors:** Ellen Stewart

**Affiliations:** ^1^ Centre for Biomedicine, Self and Society Usher Institute University of Edinburgh UK

**Keywords:** major service change, qualitative interviews, government/state, National Health Service, patient and public engagement

## Abstract

The “problem” of public resistance to hospital closure is a recurring trope in health policy debates around the world. Recent papers have argued that when it comes to major change to hospitals, “the public” cannot be persuaded by clinical evidence, and that mechanisms of public involvement are ill‐equipped to reconcile opposition with management desire for radical change. This paper presents data from in‐depth qualitative case studies of three hospital change processes in Scotland's National Health Service, including interviews with 44 members of the public. Informed by sociological accounts of both hospitals and publics as heterogeneous, shifting entities, I explore how hospitals play meaningful roles within their communities. I identify community responses to change proposals which go beyond simple opposition, including evading, engaging with and acquiescing to changes. Explicating both hospitals and the publics they serve as complex social phenomena strengthens the case for policy and practice to prioritise dialogic processes of engagement. It also demonstrates the continuing value of careful, empirical research into public perspectives on contentious healthcare issues in the context of everyday life.

## Introduction

*Building hospitals is not like building pyramids, the erection of memorials to endure to a remote posterity. We have got to get it into our heads that a hospital is like a shell, a framework to contain certain processes, and when the processes are superseded, the shell must, most probably, be scrapped and the framework dismantled*. (Enoch Powell, Minister of Health, speaking in 1961)

*[The local doctor] said what's the point of having a grand stone cross in the district when you can have a hospital?* (Public interviewee, case study 2)

These quotes illustrate a clash between treating the buildings in which healthcare is provided as mere “shells”, and creating and sustaining them as institutions with social and historical significance (Jones 2015). In recent health services research, public responses to NHS change proposals have predominantly been approached as a monolithic obstacle to progress, and policy reports have urged decision makers to “take tough decisions” (Brooks and Farrington‐Douglas 2007, Imison *et al*. 2015). Rather than question how healthcare organisations can overcome public attachment to hospitals, this paper asks how local hospitals have meaning for their communities, and what roles communities play in their change and closure. I argue that while the specific valued characteristics of hospitals vary, they can be helpfully understood not as shells within which services are provided but as socially‐constructed “anchor institutions” which hold communities together. The implication of this argument is not that hospitals should never change or close, but simply that clinicallly or managerially‐driven efforts to change them should acknowledge and offer alternative possibilities for these social roles. Efforts to understand the complexity of public perspectives on healthcare change may reveal scope for compromise, both in shaping how publics value their health services, and in helping healthcare organisations to make decisions better‐informed by the multiple “everyday” roles of healthcare facilities.

## Studies of public responses to hospital closure

References to public opposition to hospital closure are commonplace in studies of strategic healthcare change across different health systems, but there is far less empirical research *on* this perceived opposition (Cohen and Ahern 2014). In October 2017 I searched three academic databases (ASSIA, IBSS and Web of Science) for the search terms “hospital” and (closure OR disinvest* OR reconfig*). I identified 36 papers written in English which a) discussed public reactions to or engagement in hospital closure processes (excluding care homes) and b) reported empirical research. I supplemented these with papers which were not identified in the search but which met these criteria. The identified papers included a range of study designs. Despite a shared focus on the public dimensions of major change, they lacked qualitative data collection focused on public perspectives: one highly‐cited cluster of studies relies upon analysis of newspaper coverage or official documents of a handful of high‐profile change proposals including Kidderminster Hospital and St Bartholomew's Hospital (Joseph and Kearns 1996, Joseph *et al*. 2009, Moon and Brown 2001, Oborn 2008). Where qualitative data are used to explicate a public perspective on change processes, it consists of interviews with elected politicians (Fulop, Walters, Perri6, & Spurgeon, 2012) or small numbers of highly engaged individuals either campaigning against (Bryant, 2003; Taghizadeh & Lindbom, 2014) or engaging with (Abelson 2001, Droog *et al*., 2017; Farmer *et al*. 2007, Foley *et al*. 2017, Martin *et al*. 2017) change processes. The most in‐depth study of wider public perspectives on major change comes from the English NHS (Barratt *et al*. 2015). This study includes interviews modelled on “discrete choice experiments” with 20 individuals, and compares perspectives on reconfiguration of emergency care in an area undergoing change with an area which is not.

Much of this literature acknowledges a degree of heterogeneity within “the public”: Barratt *et al*. (2015) distinguish interviewees in favour of change, campaigners against change, and non‐campaigners against change. Yet their conclusion is blunt: “Our study challenges the assumption that evidence can be used to persuade communities to accommodate service reorganisations which may compromise timely access” (Barratt *et al*. 2015). The unrepresentativeness of campaigners against change also arises repeatedly within this literature (Barratt *et al*. 2015; Martin *et al*. 2017, Parkinson 2004), assuming that there exists some wider known collectivity to be represented. While a few articles identify conflicting groupings within the community (Abelson 2001, Kirouac‐Fram 2010, Pescosolido, Wright, & Kikuzawa, 2018), heterogeneity is often de‐emphasised as authors move towards conclusions which describe “the” public reaction.

Reported public responses to hospital change are overwhelmingly negative. In Table 1, I divide the reported substance of public perspectives into those presented as amenable to negotiation, and those which are presented as broadly insurmountable obstacles to involving the public in change.

**Table 1:** Public responses to healthcare change reported in identified academic studies

Perspectives amenable to negotiation or management	Perspectives not amenable to negotiation or management
Engaged rejection of financial or clinical case for change (Barnett and Barnett 2003, Oborn 2008)	Public being orchestrated/manipulated by other actors (especially hospital staff) (Abelson 2001, Dent 2003, Kirouac‐Fram 2010)
Distrust of and/or breakdown in relationship between public and authority (Abelson 2001, Brown 2003, Kirouac‐Fram 2010, Oborn 2008, Thomson *et al*. 2008)	Emotional attachment to hospitals (sometimes linked to social cleavages) (Abelson 2001, Barnett and Barnett 2003, Fontana 1988, Haas *et al*. 2001, Kirouac‐Fram 2010, Lepnurm and Lepnurm 2001, Reda 1996)
Functional fears about access to services or future viability of community (Gifford and Mullner 1988, Goyder 1999, James 1999, Kirouac‐Fram 2010, Lindbom 2014)	Symbolic role of hospitals in representing locality or wider health‐care system (Brown 2003, Gifford and Mullner 1988, James 1999, Moon and Brown 2001)
Concerns about legitimacy of process (Barnett and Barnett 2003, Daniels *et al*. 2013, Joseph and Kearns 1996, Joseph *et al*. 2009, Oborn 2008, Parkinson 2004)	Different understandings of acceptable risk (Barratt *et al*. 2015)

The perspectives reported in Table 1 suggest that the wider public both responds differently to clinical assessments of risk, and values hospital “quality” in different ways to those who plan services, leading to some pessimistic scholarly assessments of the possibility for making health service change while giving publics a meaningful voice within decisions (Barratt *et al*. 2015; Martin *et al*. 2017).

## Sociological perspectives on hospitals and publics

The approach taken in this study was informed by two bodies of broadly sociological work. Firstly, recent sociological studies of “what hospitals are” help us rethink the gap between organisational narratives of healthcare, and the everyday lived experiences of users and staff within healthcare facilities. Recent sociological studies point to the multiplicity of experience of the hospital as place beyond “the ostensible fixity of buildings” (Martin *et al*. 2015, p1011). Street and Coleman (2012, p. 6) depict hospitals as fragile, heterotopic spaces, identifying “multiple, layered, and contested orderings that configure hospitals as spaces of care, expertise, and science”. In their study, of not a hospital but a care home, Ivanova *et al*. (2016) beautifully evoke the mutability and porousness of a care home as “carescape”; making evident not only the complexity of experienced materialities, but the ways they are constituted and reconstituted through everyday use. In this study I use this more contingent sense of hospitals as assemblages to explore the contested “unmaking” of healthcare spaces, attending both to the everyday coproduction of the facilities at the centre of change processes, and to the way in which the materialities of a hospital can be experienced differently by people in different subject positions.

Secondly, social science research on publics (Braun and Schultz 2010, Felt and Fochler 2010, Mahony *et al*. 2010) explores how scientific and state actors enlist “a missing – or elusive and in some ill‐defined way problematic – public” (Wynne 2015). This literature rejects the idea that a public can be weighed and measured through the individual opinions of the individuals within it (Chilvers and Kearnes 2015, Hess 2015). This goes beyond the idea that publics interpret information based on their own prior experiences (Barratt *et al*. 2015). Rather, publics are themselves shifting collective entities, always “in‐the‐making” (Chilvers and Kearnes 2015). Seeking to integrate key insights of studies of public engagement and studies of social movements, Hess (2015) argues that we must look beyond organised events of public consultation (see also Marres 2007) towards mobilised publics, and also that we should attend to the epistemic dimensions of social movements, including their interactions with science and experts.

These existing debates have acted as “sensitising concepts” which “guide but do not command inquiry, much less commandeer it” (Charmaz 2014, p. 30) within a project which sought to understand how members of the public are involved in decisions to close or significantly change hospitals in the Scottish NHS. In this paper I ask: how can we study the involvement of “the public” in contentious decisions without reifying views which we recognise to be sociologically complex? How can we debate the future of hospitals in a way that respects the possibility of them being more than mere “shells”?

## Methods and study context

This paper draws on a qualitative study of three change projects in Scotland, with empirical data collection taking place between March 2016 and May 2018. In an effort to study a range of hospital change projects, I began case selection by assembling a total population of proposed major changes, including cases which had attracted media attention (including cases where closure was merely a rumour) and cases which had proceeded in a relatively uncontested fashion. I assembled a list of major service changes (a statutory category in Scotland) proposed between 2004 and 2014 which involved a possible hospital closure and supplemented this by searching the archives of four Scottish newspapers for “hospital + closure + NHS” within the same time period. These two processes yielded a total of 13 proposals (or rumoured proposals) to close 23 hospitals. From this I sought to identify a range of: outcome of proposal; type and size of hospital; type of community (including both rural and urban settings). I also focused on recent or ongoing closure processes, given the potential for non‐participant observation and greater ease of contacting possible interviewees. Brief summaries of the characteristics of my cases are given in box 1.

Box 1 **Case study backgrounds**

Case 1 Hospitals 1 and 2 are historic hospitals located in a rural area. At the time of the study hospital 1 offered GP acute beds, minor injuries service and some outpatient clinics. Hospital 2 offered GP acute beds and an old age psychiatric unit. The NHS Board proposed the closure of both hospitals, and the creation of a new community hospital in a growing town situated in between the other two settlements. The proposal involved inpatient beds, minor injuries service and a range of outpatient clinics being located in the new hospital. The Cabinet Secretary approved the plans, and the closures and building are underway at the time of writing. My fieldwork took place during the implementation phase of this project.
Case 2 Hospital 3 was built by subscription in the 1910s. It is in a small town in what is now the commuter belt of a city. At the time of the study the hospital offered GP acute beds, a palliative suite and minor injuries service. In the 1990s, with support from the Medical Director, an active ‘Friends of the Hospital’ group fundraised to build an extension in which daycare for the elderly could be provided. This service was closed in the 2000s. In the early 2010s, a rumour circulated that the hospital was at threat, and after a well‐attended campaign meeting the Health Board said the hospital would remain open. The Friends began fundraising to build a new, better equipped hospital, agreeing with the Board that the NHS would meet running costs once built. More recently, the Friends changed their name to ‘Friends of the Hospital and Community’, set up a number of wider wellbeing projects, and began consulting the community on different models for health and wellbeing, de‐emphasising a new hospital building as their goal. During my study, the Board began consulting on a reduction to the hospital's minor injuries function. My fieldwork took place during these two consultations.
Case 3 Hospital 4 was built in the mid‐20th Century in a socio‐economically deprived area of a large city. At the time of the study the hospital offered rehabilitation including two specialist units, a day hospital and outpatient clinics. In the late 2000s, the NHS Board proposed and consulted on its closure. After significant opposition, the Cabinet Secretary rejected the proposal. In the mid 2010s a document acquired by a local politician suggested that the Board was again considering closing the hospital. This was initially denied, but later that year the Board confirmed its intention to consult on the proposal. The Board confirmed its decision to close but the Cabinet Secretary again refused permission. My fieldwork began during the consultation, and continued beyond the Cabinet Secretary's decision.

The cases are “most different” – both in terms of their characteristics, and because my fieldwork happened at different moments within the change process. This enabled me to explore emerging explanations across different contexts, while being manageable enough to retain a sense of the context of each case (Stake 2013). At the time of the study, all the hospitals were primarily serving the elderly population: one as an acute facility, two as wholly GP‐managed community facilities, and the fourth as a joint GP/acute operation. I address this limitation of this study in the discussion section below.

My fieldwork in case studies (see Table 2) included: non‐participant observation of consultation meetings, campaign meetings and other community groups; interviews with NHS staff, local politicians and journalists (*N* = 26); and interviews with members of the public (*N* = 44). I also gathered local NHS documents relating to the proposal and consultation, along with local media and some social media (for example, Facebook and Twitter discussions on the change proposals). Interviews were audio‐recorded, and transcripts, documentary sources and my fieldnotes were analysed in NVivo using Charmaz's (2014) grounded theory approach. I began with close line‐by‐line coding with an emphasis on actions (example codes: “worrying about the big hospital”) followed by the development of progressively more thematic codes (example codes: “homely care”).

**Table 2:** Data collection

	Case study 1	Case study 2	Case study 3
Public interviewees	17	18	9
Community‐organised events observed	2	4	2
NHS‐organised events observed	0	2	1

The analysis presented in this paper is part of this wider grounded theory analysis of the full dataset Rather than the organisational approaches to change and consultation described in interviews with staff, I concentrate in this paper on my interviews with members of the public, in order to explore the specific question of community responses to hospital change. However my interpretations of these responses developed iteratively during data collection. I make several references to other data (an official document, fieldnotes from an event and one staff interview) where necessary to support specific argument. My choice of interviews as method allowed individuals to reflect on their own views and experiences of the changing hospitals (Brinkmann and Kvale 2014). Alternatives such as focus groups would, while potentially allowing me to engage higher numbers of participants, have created a group discussion on a topic where a vision of local “public opinion” was already defined and expressed in media coverage. While relevant, this type of data was captured through observation of community events and analysis of reports of consultation events. Interviews were an attempt to disaggregate and explore “public” views. This choice brought other risks, specifically in requiring people to discuss an issue which may be fairly peripheral to their lives (Eliasoph 1998, Stewart 2015), but my conversational approach to the interview sought to make space for interviewees to express ambivalent or negative views about the local hospitals. Interview questions covered the interviewee's background, time living in the community, their experiences of and views on the hospital, and the consultation and/or campaign around proposed changes.

The choice of “public” interviewees involves engaging with long‐standing debates in this field about terminology (patient, citizen, service user, community member) and about who has a legitimate right to a say over healthcare decisions (Lehoux *et al*. 2012). My approach was simply to seek out residents of the area willing to be interviewed, looking to ensure a mixture of people who had been closely involved with the change process, and people who had not. Interviewees were recruited through NHS staff (approaching individuals who had sat on stakeholder groups for the project or similar), and through campaigners (identified via local media or social media, or sometimes through NHS staff). None of my public interviewees were current staff members of the local NHS organisation, although a significant number of retired interviewees (12) had previously worked in either the NHS or social care. I also actively sought out interviewees who had not engaged with the consultation or campaign via snowballing, contacting community councils, chatting to people in social spaces such as coffee shops, and social contacts. These interviews helped to contextualise the perspectives of currently actively involved members of the public: I make no claims as to how prevalent these views were in the wider community. In acknowledgement of the “sociological concreteness” (Lehoux *et al*. 2012) of my participants, I do not attempt to distinguish the data from people who were, at the time of our interview, positioned differently on the question of the change. Twelve interviewees were male and 32 female. The age of my interviewees ranged from 30s to 90s; 25 interviewees were retired. Recruitment was more challenging in case study 3, which I attribute partly to the urban setting (compared to the ease of snowballing in semi‐rural areas) and also to heightened political tension around the proposal causing unease from potential interviewees who were not strongly aligned to one position. For example, one slightly reluctant interviewee opened the door shouting “I'm too old to be banging a drum about this” (fieldnote excerpt CS3).

## Findings

My research sought to explore both public feelings about threatened hospitals and public decisions about taking action. These are not independent phenomena (Wagenaar 2011). Existing studies in this area have focused either on observed public behaviour (invited or uninvited) or on what members of the public “think” about change. Borrowing from Yanow's (1996) interpretive framing “*how* does a policy mean?”, this paper asks “how does a hospital mean?” to emphasise the practices through which hospitals have meaning in communities. While I was initially inspired by classic accounts of oppositional “contentious politics” (Della Porta 2013, Tilly and Tarrow 2006), prolonged interpretive case studies allowed me to recognise a much wider range of public actions around hospitals. This section sets out how interviewees described what hospitals “meant” to them, followed by the range of public action evident in these change processes. Meaning and action were not merely heterogeneous within each case study, but also co‐constituted; what was valued about these hospitals was in a recursive relationship with forms of public action. That is to say, when a change was proposed, consultation and mobilisation processes not only reflected public feeling for hospitals but created, shifted or strengthened it. In parallel, public feeling for hospitals influenced the likelihood of change being proposed.

### What hospitals mean

The hospitals fulfilled a range of roles in their communities, but the way public interviewees described their value rarely aligned with organisational descriptions of their functions in official documents. In case study three, an official report on the consultation explicitly described these differences in understandings: “There were misunderstandings in a number of submissions about the current function of [hospital 4], including that the hospital provides care for patients discharged from acute services and that it is a local hospital.” All four hospitals were predominantly providing care for elderly patients, some with a minor injuries role for the wider community. In each case with a formal change proposal, it was proposed that current services would be moved into a new or existing alternative location, care‐at‐home, or “step up step down” beds in local care homes. Public interviewees rarely described their objections in terms of the NHS's clinical rationales for change (which included technological developments, disability access and patient safety). This likely reflects the general and community functions of the facilities in the study, and it contrasts with examples such as emergency care and maternity care, where contending definitions of acceptable medical risk have been identified (Barratt *et al*. 2015, Farmer *et al*. 2007). However, I suggest it also stems from this study's inclusion of people who had not engaged with the consultation process, and of the open question in each interview: “what, if anything, does this hospital mean to you?” This created space for the “complex everyday configurations and alignments of materialities, social practices and representations through which particular spaces are constituted as hospitals” (Street and Coleman 2012, p. 6).

Many interviewees described the hospitals as somehow special. Interviewees described moments of kindness which were intensely local – a patient given a cup of tea in a mug branded with her local church's name; a patient's daughter being let in to visit her mother at 2am because she couldn't sleep – but which also related to the relative quietness of these hospitals compared to the alternative tertiary facilities. The relatively low bed occupancy simultaneously justified closing hospitals while creating a rehabilitative environment patients valued. Describing a parent's experiences of being nursed locally, this interviewee compared it to an earlier stay in the closest tertiary hospital:

Just that there was, that the nurses had more time to care, really, I think. It wasn't the, it certainly wasn't the facilities, or the building, it was just the standard of care from the staff, and that they had the time to do their jobs, really. (P8.CS2)

This interviewee explicitly denies the significance of the building to emphasise particular qualities of care possible within these smaller, quieter facilities. The physical environments of several of the hospitals were often acknowledged to be inadequate, but this was usually blamed on NHS management, rather than intrinsic deficiencies of the building. For at least some of the interviewees, the problems of the fabric of the building did not dampen their attachment to it; summing up hospital 3 one interviewee stated “*it's minging* 1 *, but it's ours*” (P3.CS3).

Hospitals were also valued for reasons unrelated to healthcare: for example, because they created stable, relatively well‐paid local jobs. When asked about what the hospital meant locally one interviewee specifically emphasised the importance of stable public sector jobs in an otherwise seasonal economy.

Good well‐paid jobs that are not in the hospitality sector; I think that will be a big miss to the community. (P17.CS1)

This was particularly salient in case studies 1 and 3, due to their local job markets, although the promise that there would be no redundancies was cited as reassuring. Hospitals were also valued as a keystone of the community. This felt particularly significant alongside a general decline in services (from public toilets to primary schools). In the following extract, one interviewee described the hospital as one of a range of closing or reduced amenities which offered scope specifically for social interaction:

I see everything falling into place with the hospital. You know, as I say I don't see a hospital just as a building in its entirety, it's everything that goes on in a hospital and so I felt that the Post Office, it relocating … and, you know, it's going down to a wee shop. I don't want to sound disparaging but, you know, you would pass that and never give it a blink, but the Post Office had a presence. The hospital's got a presence and even the recycling place – at least there's something on a Saturday and Sunday, a place of social interaction because it's not open during the week so people go down there. I actually felt that, you know, it was creating a … for me, and I am using the word bereavement, but this is going to cause a real bereavement. (P3.CS1)

This poignant description of a hospital's significance in a town in decline is easy to dismiss from a technocratic perspective which seeks measurable health outcomes. And yet, this “presence” was a recurrent part of what interviewees valued about hospitals.

These statements of specialness and value did not consistently correlate to opposition to closure. While only one interviewee was actively glad that the hospital would close (seeing it as a waste of money), multiple interviewees who described their attachment to these hospitals, could also see advantages to the change. Describing her response to letters against the proposal in the local newspaper, one interviewee stated:

The good thing is [in the new hospital the rooms are] going to have their own toilets and that's my temptation to write to the [newspaper], every time somebody says “we don't need a new hospital, we want to keep [hospital 1], we want to keep [hospital 2]”. They're focusing on the building rather than the care. (P10.CS1)

For another interviewee, the hospital offered services which were limited both in scope and in relevance to the wider population:

If I'm honest, there's a lot of people in the community, to which the hospital really means nothing, because the actual hospital offers them nothing. Because it's generally, just really elderly people, and unless you've got elderly relatives, it doesn't mean a lot to you … And even then, you know, they don't necessarily have any link to the hospital. Because, you know, people, it's not the way things are going now. You go into [closest tertiary hospital], and it's all about rehabilitation, and getting you home as quickly as possible. (P8.CS2)

While there was implacable opposition from some interviewees, at least some interviewees in each case study were supportive of proposed changes to their hospitals.

### What publics do

My analysis identified four overlapping forms of public action around threatened hospitals: engaging, evading, fighting, and acquiescing to change. Cooperative *engagement* between members of the public and local NHS managers was particularly evident in case study 1. Here, NHS managers had painstakingly built cooperative relationships with key members of the community. Outreach work (Stewart 2016) from NHS staff in attending local meetings which were happening anyway (community councils, Friends of Hospitals, local community charities) had built trust and dialogue. Within this, members of the public described engaging to establish shared understandings of both current models and potential alternatives. In one town a public interviewee was credited with having *proposed* the closure of the hospital, and in another, key groups were amenable to the idea as a means of protecting the wider availability of local services. This sort of relationship can be interpreted as “complicity” (Martin *et al*. 2017) but this does disservice to the strategic work in which community members were engaged. More than one interviewee stated that they were staying involved beyond the closure of the hospital to try to influence decisions about new provision of care, and to “keep an eye” on promises already made. Explaining his continued involvement with NHS project groups beyond the decision to close the hospital; one interviewee described the need to make sure new services met local needs: “*It's still all to fight for”* (P13.CS1).

My research also revealed ongoing, everyday work within communities to support, strengthen and defend local hospitals, including significant fundraising and volunteering. I characterise these as evasive actions, in that they were ongoing in parallel to NHS organisational attempts to make changes to the hospitals. They included voluntaristic service improvement work focused on patient experience; tea trolleys, entertainment and a gardening project, for example. This work also evaded closure by building visible support around the hospital. This interviewee described the role of a “Friends of the Hospital” group's fundraising and support in the NHS's decision not to pursue a previous proposal to close the hospital.

We were showing ourselves to be politically very effective and I've got no doubt that was a factor in the equation. We'd also shown ourselves generally as a community campaigning group to have a lot of support which is well evidenced, you know, with the number of collections there are at funerals, the number of people that turn up for our events, the number of legacies that are left to us. (P3.CS2)

While this quote points to the efficacy of discursively framing a hospital as widely‐supported within the community, evading closure was not always an explicit goal. Rather, community building of “care infrastructures” (Langstrup 2013) was often performed in gratitude for one's own, or a loved one's care, or in anticipation of future care within the hospital. These structures of support did, however, create opportunities for easy mobilisation, and facilitated the monitoring and pre‐empting of local NHS plans for the hospital. This included encouraging recent patients to submit favourable online reviews of the hospital (case study 3), and convening (and publicising) public meetings in rapid response to any rumours of closure (case studies 2 and 3). Such meetings, usually absent a speaker from the NHS given the unconfirmed status of rumours of closure, acted as a show of strength from the community. In my fieldnotes following one protest meeting I tried to capture the sense of energy in the room:

Busy meeting on a cold night (hard frost). Standing room only … Repeated calls of “We will fight this. We fought it last time”… Really lively atmosphere – every speaker/question applauded. (fieldnote excerpt CS 3)

Such events were facilitated by existing structures of support for hospitals, including both Friends of the Hospital groups and patient‐led support groups.

My research identified a range of approaches to *fighting* a proposal for change once it was formally announced. Once the members of a community organised, often facilitated by an existing group, dialogue between the NHS and the campaign became largely strategic, often conducted in public through newspapers and social media. Tactics were chosen carefully. In one case study site, a ‘Hospital Action Group’ notionally broke off from the Friends of the Hospital group in order to engage more effectively in campaigning and political negotiation. Describing the decision, one group member explained:

There was a bit of overlap but it was meant to make things easier. It took any of the sort of horrible jobs away from the Friends, that was the theory, you know, the really getting on people's nerves and saying the wrong things, so they were quite forceful. But eventually we merged them back into the Friends. (P1.CS2)

In another, two fairly distinct campaign groups sprang up, broadly affiliated along party political lines and working independently of each other.

Petitions and marches – mainstays of newspaper coverage of hospital closures – had been used in previous campaigns in all three case study sites. Remarking proudly on a large‐scale previous campaign, one interviewee described his confidence that the campaign had demonstrable wider support.

We had the marches for [hospital 4], the protests outside parliament, you know, we got the community involved, the petition signatures raised eventually 14,000 people … So that side, you know you're doing it for the community. (P1.CS3)

The work of fighting a closure was demanding, especially for people in poor health. Active campaigning tended to bypass the formal consultation process, and some NHS staff expressed suspicion about whether it represented “ordinary” public views. However, its key function was in strategically amplifying those views; demonstrating (ideally quantifiable) heft in contrast to Lehoux *et al*.'s (2012) “unbearable lightness of citizens”. Campaigning reflected and generated feelings of solidarity among concerned publics, and in so doing sometimes precluded compromise or dialogue with management.

Another strategy for fighting proposals was evident in case study 3, where campaigners bypassed NHS managers in favour of the elected politicians to whom they are accountable. Meetings with councillors, Members of the Scottish Parliament or Members of Parliament were commonplace across all three case studies. In some cases, representatives would simply offer some advice to the campaigners, even, as this interviewee described, seeking to manage their expectations of the possibilities of overturning a decision.

I explained a lot of this isn't democracy, no public meeting and everything else, [Member of Scottish Parliament] then said “I think your only avenue is a judicial review”. Now, in all seriousness, when a politician is telling a rural community to seek a judicial review first of all the cost, second is the time. Bloody hell. (P9.CS1)

This interviewee understood the role of their elected politician was to take up their concerns and act on their behalf, and expressed frustration that the meeting had not prompted that action.

In others, politicians were more closely involved. In previous campaigns in case study 2, the local MSP was quoted in media coverage offering to mediate between NHS staff and the community. Sometimes, “going the political route” was necessarily secretive, and discrepancies would develop between what interviewees were willing to say in interviews, and what they would describe to me afterwards, “off the record”. Relying too much on representatives could backfire, as when one very active politician was apparently pulled into line by his party and stepped back suddenly from a campaign he had been actively “fronting”. My interest here is the relationship between the image of campaigns in media coverage and the practices occurring on the ground. At times, when campaigns were working closely with elected representatives, they were entirely invisible to conventional measures of community opposition such as media and social media coverage, and protest marches.

Not everyone fighting a proposal was part of an organised campaign. In case study 1, where the NHS's “pre‐engagement” work to create dialogue had been most thorough, key community actors were broadly onside with the proposals, and no coherent campaign grouping formed. Instead, a small number of individuals, with a firm belief that they represented wider community opinion, engaged in something akin to resistance tactics; pointing out flaws in NHS statements, and writing individual letters to newspapers or politicians. No shortage of work went into this campaigning activity, yet these individuals avoided any wider mobilisational work. When asked about working with other campaigners, one interviewee firmly rejected the idea:

No, I'm an individual, I'm very much an individual. My professional life was very much being an individual … I've just, as I said, voiced my opinions as an ordinary citizen. I think it's morally and fundamentally wrong and there's an agenda which has been hidden from the public which is essentially about raising money. (P7.CS1)

This was not because individual campaigners were less committed to their cause; rather, it was a question of preference, even personality and a discomfort with collective organising.

Finally, in each case study I sought out small numbers of interviewees who had engaged with neither the consultation process nor the campaign. While I characterise this as *acquiescing* in service change, not taking action was, of course, rarely presented as a decision taken in these interviews (Stewart 2015). Interviewees who were not closely involved in debates about the hospitals were often unclear whether and how they might have contributed to past consultations or campaigns. Reflecting on her engagement with a previous campaign around the hospital, one interviewee mused:

I would have signed a petition, probably. I don't think I would have gone out on the streets. (P13.CS2)

In other cases, people expressed some sadness at the potential loss of a local hospital, but described their own priorities for taking action as being focused elsewhere, for example on education or leisure services. In the following discussion, two interviewees compared their relative inaction on hospital changes to their quick response to changes to school provision:

“Int: Would you be more active, on your high horse as you say, about those issues because you care more or because they feel like less of a fait accompli?

RES1: Just they're closer to us aren't they?

INT: More relevant?

RES1: Yeah.

RES2: But I also felt like we were part of a body that could actually do something about it because there was a body of parents who felt like you could.” (P4.CS1 & P12.CS1)

As this quote indicates, the combination of caring about a service with existing groups this interviewee felt “part of” and able to act within, was crucial in shaping decisions about taking action. This was as true for members of community groupings around hospitals, who often referred to belonging and the importance of a sense of shared purpose. There was a rough generational divide here, with younger interviewees somewhat less likely to take action to defend a hospital. However, this difference between people who actively engaged, evaded or fought a proposal, and those who “acquiesced”, was rooted in their social worlds and their familiarity with the facility in question, rather than in a demographic characteristic. Views on the proposal developed in discussion (or non‐discussion) with friends and acquaintances, which also created and shaped opportunities for action.

## Discussion

This paper contributes to our understanding of community responses to hospital change not by presenting a fixed “public opinion” on the topic (whether ripe to be tackled or stubbornly intransigent) but by exploring the edges and intersections of “hospital” and “community” as social phenomena. My three case studies were different both in terms of the type of community and the mix of services within the changing hospitals. However, in all three these were interconnected: communities were shaping the hospitals and hospitals (perhaps especially when threatened) offered a locus around which communities cohered.

A cluster of existing studies of hospital closures (Brown 2003, Joseph *et al*. 2009, Kearns and Joseph 1997, Moon and Brown 2001) have argued for the wider social significance of hospitals for communities from the perspective of cultural geography. These wider meanings are neglected in more recent studies which narrowly focus on mechanistic public responses to given system rationales for closure. However, this group of cultural geography studies is dominated by discourse analysis implicitly “woven from local sources” (Kearns and Joseph 1997). The richness of the data reported in this present paper contextualises the “hospital as employer” or “hospital as symbol of community” within everyday social practices which care for and construct the hospital.

Recent sociological studies of healthcare facilities (Ivanova *et al*. 2016, Jones 2015; D. Martin *et al*. 2015) illuminate the porous boundaries of hospitals. This porousness – the hospital as assemblage – is crucial to reconceptualising public “responses” to proposals for change. For many of my interviewees, hospitals were defended neither in a consumeristic demand for particular clinical services nor a rejection of the clinical framings of proposals. Interviewees more commonly described the familiar, high‐quality care within the hospitals, and talked about them as assets which the community had (in some cases) created and (in all cases) helped to shape. Hospitals thus pulled together multiple strands of community life. These public understandings of value echo elements of models proposed by experts around hospitals as “anchor institutions” in communities (Norris and Howard 2015) or “social infrastructure” (Klinenberg 2018), and recognise the recursive relationship between community wellbeing and spaces for community elaborated in Warin *et al*.'s (2000) Australian study of women's health centres or Farmer *et al*.'s (2012) capitals framework. New models of care ‐ care‐at‐home services, services dispersed across multiple sites or “intermediate care” beds in a care home – were often perceived as *un*anchored; less locally‐embedded, and also easily removed or reduced.

Exploring public responses to hospital closures sociologically entails beginning with public framings of the issue at stake, and respecting those framings (Marres 2007, Wynne 2015). Health policy commentators often complain that the public is excessively focused on hospitals as physical buildings. For example, Jeremy Taylor of the charity National Voices states “we have a public discourse about healthcare that invests too much emotional baggage in bricks and mortar” (quoted in Ramesh 2010). In 2016, NHS England's national clinical director for innovation argued provocatively (and, one assumes, hypothetically) “Do we need to blow up all our hospitals and start again?” (quoted in Payne 2016). Such arguments are only likely to gain traction as technology extends “care infrastructures” outwards into the home (Langstrup 2013). Innovation‐focused narratives – using a notional blank slate to redesign health systems – must do more to understand how and why communities value, and often defend, their hospitals. Hospital buildings are created, maintained and defended as talismans of the shifting promise of care within that locale. Beyond any specific service delivered within the walls, hospitals are enacted as a shared public commitment to care which, in the case studies reported here, are to a significant extent a collective achievement, not a gift from the NHS to the population.

The temporal dimension of these contestations is important (Pollitt, 2008). The rupture of a closure was situated within a community memory which outlasted that of most current NHS managers. My interviewees emphasised their perception that the threat of closure was only ever postponed. One interviewee, describing the end of a previous attempt to change services within the hospital, pinpointed NHS investment in maintenance as a sign of temporary safety for the hospital.

The day we knew that the circumstances had changed in terms of the place possibly being closed was the day they started painting the windows. They started doing some maintenance on it, so we knew it was safe, for a while. (P3.CS2)

The legacies of distrust from previous, sometimes poorly‐planned and unexpected, attempts to close hospitals coloured how communities responded to new attempts. Proposals focused on future promises and rarely acknowledged that previous reforms had been based on now‐rejected rationales. Telling me about a previous closure of an innovative day care service for elderly people, this interviewee described how NHS staff had denied the possibility of a now‐dominant policy agenda.

I can remember being at meetings, and them saying, you know, that's social care, it's nothing to do with us, we're not doing it anymore. And of course, now, it's all about integrating health and social care. But then … and I can remember sitting at the meeting and saying ‘but you can't separate health and social care’. But ten years ago, you could. Now, you can't, but you could then. (P8.CS2)

In their work in New Zealand, Joseph *et al*. (2009) argue that “the protracted nature of the “real” as opposed to the “formal” closure process served to dilute and deflect public debate (and media interest) in the demise of these previously prominent institutions”. However, interviewing residents renders visible the cyclical nature of recurrent threats, and perceptions of NHS neglect of unwanted buildings in between active attempts to close them. While debates did indeed disappear from media coverage for years or even decades, communities continued to debate and crucially to actively construct (via fundraising, volunteering and defending) these hospitals in between “real” closures.

Literature on public involvement in healthcare often questions the representativeness of public actors (Barratt *et al*. 2015, Harrison and Mort 1998; Martin *et al*. 2017). When a hospital closure is proposed, both campaign groups and healthcare organisations seek, through protest and consultation processes, to invoke public opinion and present it as broadly unified (Osborne and Rose 1999). Interviewing a range of residents of the hospital “catchments”, including but also going beyond those actively engaged in either opposing or influencing change, shed light on these contested representations of identity and legitimacy. Employing sociological understandings of publics as constructions mobilised and invoked guards against an imagined public which is homogenous in its views and simply represented, rather than formed, through action. Grouping observed responses by activities, rather than by types of people, is a deliberate sociological strategy to recognise the unpredictability and variability of human action (Becker 1998, Hacking 2007). Some of my interviewees were simultaneously fighting, engaging with, and evading proposals, and many had moved through different phases of activity over the years. Others were undertaking what I interpret as “evasive” volunteering activities while broadly acquiescing to management proposals to close the hospital. Abu‐Lughod (1990) entreats us not to romanticise resistance to seemingly hegemonic power but rather to follow it as a diagnostic of power. Tracing the work of communities around threatened hospitals is sociologically intriguing in its own right, but also reveals the fragility of community empowerment within a health system still dominated by both medical and managerial rationalities.

A key limitation of this study relates to the focus on community and rehabilitative facilities, rather than, for example, emergency or maternity care. In practice, such tidy distinctions are complicated by a focus on hospitals as complex social institutions (Street and Coleman 2012) with histories and sometimes contested perceptions of their current use. Two of four hospitals in this study still had a minor injuries function; another seemed to informally fulfil this function for the community; and the fourth had offered this service in the last decade. None of the hospitals currently offered maternity services, but two had in living memory. Varied understandings of the past and present roles of these hospitals were central to contestation over their futures. Organisational attempts to shift the debate towards alternative organising concepts like “patient pathways” (Allen 2009) were often resisted by campaigners. Nonetheless, the broad type of hospitals examined in this study likely colours the extent to which communities emphasised their non‐medical value. In this study, campaigners tended to argue for the salience of wider social and community factors alongside clinical rationales for change. Their descriptions rarely emphasised an engaged rejection of that clinical case, while this is a central finding of Barratt *et al*.'s (2015) study of emergency care. Further research should seek to identify whether this difference is intrinsic to different types of hospital, or generated by divergent study designs: Barratt *et al*.'s use of discrete choice experiments means that research participants were directed to “trade‐off” pre‐defined characteristics of clinical care. My findings echo earlier findings from geography studies (Brown 2003, Moon and Brown 2001) which have been relatively neglected as researchers have focused on healthcare change as a policy problem, not a social phenomenon. In orienting towards community practices of care, I supplement the focus on textual and linguistic representations of hospitals in the cultural geography literature. Within the context of renewed policy interest in public responses to healthcare change, this article seeks to bring a more nuanced sociological sense of what publics are (and do) to existing insights around the co‐constitution of communities and healthcare facilities.

## Conclusion: publics and their hospitals

Recent studies have painted a bleak picture of the chasm between public and health system perspectives on major change to hospitals (Barratt *et al*. 2015; Martin *et al*. 2017). This paper turns a sociological lens on the topic, explicating more complex, and potentially more hopeful dynamics. Change proposals intervene into ongoing relationships between hospitals and their communities, in which community practices of care for hospitals are “constituting matter, both in the evidential sense of bringing matter into being and in the sense of constituting what ought to matter” (Woolgar & Neyland, 2013). The flexibility and longer timescales of the interpretive case studies reported here, as well as the inclusion of case studies beyond those which make national news, enable us to understand system‐led projects of change in context. Within this, we can recognise the complexity and context‐dependence of what a hospital “means” (Jones 2015) and the possibility of engaging otherwise with a community's care for and defence of those meanings. Specifically, if neither publics nor hospitals are self‐evident, known entities, then engagement processes must first listen, in order to understand the gamut of community perspectives on hospitals, including non‐medical concerns. Respectful listening builds trust, and contributes vital information to inform organisational plans for change. Other research has demonstrated that an organisational sense of urgency can crowd out the possibilities for public influence over service change (Martin *et al*. 2017). This paper concurs that thorough engagement needs time. More substantively it needs to go beyond testing public appetite for clinical rationales towards understanding the lived experience of services in their communities. Recognising that communities have relevant knowledge of their carescapes beyond patient experience of treatment (Kearns 1993) requires a more “diffused and democratic” ethos in health system decisionmaking (Cribb 2018). In other co‐authored work I explore the difficulties in achieving such shifts via policy tools alone (Stewart *et al*. 2019). Reframing planned engagement as an opportunity for organisations to learn from their publics requires wider recognition of health and medicine as collective endeavours (Cribb 2018).

While a normative case is sometimes made for the right of communities to influence major service change, debates (at least in the UK) rarely acknowledge that a community's commitment to its hospital could be understood as an asset, and not an obstacle, to the provision of high‐quality healthcare. The assumption of non‐negotiable public opposition to healthcare change may actually widen the gap in perspective between the various protagonists: Foley *et al*. (2017, p. 804) report one manager admitting that they avoided public engagement because “we wouldn't have got agreement anyway”. The range of public perspectives expressed in these case studies showcased significant experiential knowledge about health and wellbeing in local contexts, and refutes the inevitability of public opposition to change. This knowledge was not all consistently or radically new to those planning change, but dialogue became impossible once communities felt attacked. That the hospitals concerned *mattered* in different ways to different people – as a locus of “presence”, as an infrastructure of community care, as a marker of a community's past and future – limits our ability to draw firm generalisable conclusions. However, this contains the nub of a finding with translational relevance. Hospitals are neither shells for service delivery nor mere symbols; they do other things in communities. Studies which seek to isolate clinical consequences of these complex social systems, or to identify a fixed public opinion on those clinical consequences neglect that both publics, and hospitals, are social phenomena; multi‐faceted, shifting, and in process (Massey 1994).

## Note

1 Scottish colloquial word meaning smelly, ugly or unattractive.
